# Different surgical methods of hysterectomy for the management of endometrial cancer: a systematic review and network meta-analysis

**DOI:** 10.3389/fonc.2024.1524991

**Published:** 2025-01-15

**Authors:** Yuquan Yuan, Qin Tan, Yingfan Chen, Keyang Zhu, Bin Pan, Bao Liu, Chunyan Ren, Ganghui Li, Cheng Chen, Chengzhi Zhao

**Affiliations:** ^1^ Department of Gynecologic Oncology, Chongqing Health Center for Women and Children, Chongqing, China; ^2^ Department of Gynecologic Oncology, Women and Children’s Hospital of Chongqing Medical University, Chongqing, China; ^3^ Department of Neonatology, Chongqing General Hospital, Chongqing University, Chongqing, China; ^4^ Chongqing General Hospital, Chongqing University, Chongqing, China; ^5^ Clinical Medical College, North Sichuan Medical College, Nanchong, Sichuan, China

**Keywords:** endometrial cancer, hysterectomy, meta-analysis, systematic review, surgical complications, prognosis

## Abstract

**Background:**

Emerging surgical methods are utilized to treat endometrial cancer. The study aimed to assess the efficacy and safety of four common surgical methods of hysterectomy.

**Methods:**

We systematically searched the PubMed, Cochrane Library databases, Medline, EMBASE and Web of Science from their inception until April 30, 2024. We used hazard ratios (HR) for overall survival (OS) and disease-free (DFS), odds ratios (OR) for categorical outcomes, and mean differences (MD) for continuous outcomes with 95% confidence intervals. These were pooled in Bayesian network meta-analysis models. The surface under the cumulative ranking curve (SUCRA) was used to illuminate the probability that each method would be the best for each outcome.

**Results:**

Thirty studies comprising 13446 patients were included. Robotic hysterectomy (RH) retrieved fewer pelvic lymph nodes than open hysterectomy (OH). OH showed a significantly higher postoperative complication rate than laparoscopic hysterectomy (LH) and RH. LH had a higher intraoperative complication rate than OH. According to SUCRA values, OH ranked the highest in the number of retrieved pelvic lymph nodes (0.89), intraoperative complications (0.73), and operative time (0.97). LH ranked the highest in DFS (0.81) and overall survival (OS) (0.87). RH ranked the highest in the postoperative complications (0.95). Laparoscopic-assisted vaginal hysterectomy (LAVH) ranked the highest in number of retrieved para-aortic lymph nodes (0.72).

**Conclusions:**

There are no significant differences among the four surgical methods in DFS or OS. The use of uterine manipulators does not affect prognosis. OH is the best method for shortening operative time, dissecting the pelvic lymph nodes and controlling intraoperative complications. LH and LAVH have an advantage in para-aortic lymph node dissection. Besides, LH has the best advantage in DFS and OS. RH has advantages in controlling surgical complications.

**Systematic review registration:**

https://www.crd.york.ac.uk/PROSPERO/, identifier CRD42024529974.

## Introduction

Endometrial cancer (EC) is the sixth most common cancer among women, with an increasing incidence worldwide (1). Surgery is the primary method for diagnosis and treatment of EC (2–4). There are several surgical methods for treating EC, including traditional open hysterectomy (OH), laparoscopic hysterectomy (LH), robotic hysterectomy (RH), and laparoscopic-assisted vaginal hysterectomy (LAVH). Although minimally invasive approaches such as LH, RH, and LAVH have recently gained popularity among surgeons, there is still controversy in the choice of surgical method (3–5). Therefore, analyzing and comparing the efficacy and safety of different surgical methods is crucial for developing individualized surgical strategies.

As a traditional surgical method, OH is performed through an abdominal incision under direct visual observation, resulting in a shorter operation time (6). However, OH has some drawbacks, such as significant trauma, longer postoperative recovery time, noticeable pain, and a higher rate of postoperative complications. In recent years, minimally invasive surgery has rapidly developed and has become the preferred method for treating early-stage EC (7–10). Adekanmbi et al. reported that over an 8-year period, the use of OH in the United States decreased significantly from 42.1% to 16.7% (11). LH is performed by surgeons using surgical instruments for remote manipulation and is associated with less trauma and fewer postoperative complications. Additionally, it has been demonstrated that the oncological outcomes of LH are comparable to those of OH (6). However, the procedure of LH may be limited by the manipulation of instruments, particularly in obese patients (12). RH can overcome the limitations of LH because its unique mechanical arms and 3D visual field enable precise operations. However, the high cost of RH prevents its wide application (13, 14). LAVH is a combination of vaginal hysterectomy and laparoscopic techniques, allowing for a comprehensive and direct assessment of both the uterus and the intra-abdominal (15). Unlike LH, part of the surgical procedure for LAVH must be performed through the vagina (16). Some studies suggested that it may not be suitable for patients with an excessively large uterus or restricted uterine mobility (17, 18).

Uterine manipulators are widely used in LH, RH, and LAVH to expose the spaces around the uterus. However, the use of uterine manipulators may increase the risk of tumor spillage, raising concerns about minimally invasive approaches among patients (19, 20). Although some studies suggested that uterine manipulators did not affect oncological outcomes, they overlooked the impact of differences between minimally invasive surgical approaches (such as LH and LAVH) on oncological results (21, 22). Moreover, previous meta-analyses focused on pairwise comparisons between two surgical methods (23, 24). However, no comprehensive network meta-analysis (NMA) has compared the four surgical methods for EC.

This systematic review and NMA aimed to compare the efficacy and safety of different surgical methods and explore the potential impact of uterine manipulators on prognosis. Additionally, the study quantitatively ranked all outcome indicators to clarify the advantages and limitations of each surgical approach, providing a reference for the rational selection of surgical methods.

## Methods

This system review and meta-analysis was conducted in alignment with PRISMA (Preferred Reporting Items for Systematic Reviews and Meta-Analyses) and AMSTAR (Assessing the methodological quality of systematic reviews) Guideline (25, 26). The Meta-analysis of Observational Studies in Epidemiology (MOOSE) Checklist was used to abstract data and assess data quality and validity (27). This study has been registered in the International Prospective Register of Systematic Reviews (ID: CRD42024529974).

### Eligibility criteria, information sources, search strategy

We conducted a systematic literature search of PubMed, Cochrane Library databases, Medline, EMBASE and Web of Science published up to April 30, 2024 to find all relevant studies. The Medical and MeSH terms and their combinations were searched in the [Title/Abstract]: endometrial neoplasm, endometrial carcinoma, endometrial cancer, hysterectomy, abdominal hysterectomy, open hysterectomy, conventional hysterectomy, vaginal hysterectomy, laparoscopy-assisted vaginal hysterectomy, laparoscopic hysterectomy, endoscopic hysterectomy, minimally invasive surgery, Robotic hysterectomy, Da Vinci surgical system, robot assisted hysterectomy, robotic-assisted hysterectomy. The detailed search strategy was shown in **Supplementary** Search terms.

In addition, reference lists of the retrieved articles were checked to identify other eligible studies.

Two researchers carried out the literature search separately, and any disagreements were solved by consensus. The abstracts and titles of the obtained studies were checked and excluded if considered unrelated. The full text was reviewed to determine the final articles that were included. Any discrepancies were settled through discussion with a third reviewer.

### Inclusion and exclusion criteria

Due to the high costs of robotic surgery and other reasons, there is currently a lack of randomized controlled trials (RCTs) on RH. To comprehensively discuss the strengths of the four kinds of hysterectomy, we included a portion of high-quality observational studies on RH, following a rigorous Newcastle-Ottawa Scale (NOS) quality assessment (28). To reduce potential confounding factors, only RCTs were included for other surgical methods (OH, LH, and LAVH) besides RH.

Studies were selected according to the following inclusion criteria (1): Study design: RCT or High-quality observational study (detailed description above). (2) Population: patients with EC who have undergone hysterectomy. (3) Intervention: studies comparing at least two kinds of hysterectomy, including OH, LH, RH, and LAVH, and the surgical scope includes a hysterectomy combined with bilateral salpingo-oophorectomy and selective pelvic/para-aortic lymphadenectomy. (4) Outcomes: studies reporting at least one outcome of interest are mentioned below. (5) Language: studies that were published in English. The exclusion criteria were: (1) Reviews, Case reports, Letters to the editor, etc. (2) Low-quality studies (NOS score < 7). (3) Duplicate articles.

### Data extraction

Two researchers independently evaluated the full texts, assessed the quality, and extracted data. If discrepancies arose, they were resolved by consulting a third reviewer and thoroughly comparing the data. The extracted data mainly included the following: (1) Study information: first author, publication years, countries, and study design. (2) Baseline characteristics: age, body mass index, tumor staging, tumor grading, surgical methods. (3) Outcome information: disease-free survival (DFS), overall survival (OS), intraoperative complications, postoperative complications, number of retrieved pelvic lymph nodes, number of retrieved para-aortic lymph nodes and operative time. Intraoperative complications mainly include ureteral injury, bladder injury, bowel injury, vaginal injury, pelvic nerve injury, uterine rupture, subcutaneous emphysema, and hemorrhage. Postoperative complications mainly include urinary tract infections, bladder dysfunction, bowel obstruction, deep vein thrombosis, pulmonary embolism, wound infection, and lymphedema.

Survival outcomes in the original articles included hazard ratio (HR) and 95% CIs for DFS and OS. If these variables were not available explicitly, the Engauge Digitizer v4.1 software was applied to obtain data from the Kaplan-Meier survival curves according to the method of Tierney et al. (29). The natural logarithm of HR (InHR) and SE (standard error) were calculated for subsequent analysis.

### Risk of bias assessment

Two independent reviewers evaluated the RCTs by the version 2 of the Cochrane risk-of-bias tool for randomized trials (RoB 2). The methodological quality of the observational research was assessed by NOS. Three broad subscales included the study group selection, group comparability, and exposure and outcome elucidation. Only observational research with an NOS score of at least seven can be included in this study.

### Statistics

#### Pairwise meta-analysis

The pairwise meta-analyses (PMA) were conducted for direct comparisons reported at least twice using STATA statistical software (version 14.0; StataCorp, College Station, TX) (30). Continuous data were measured using the mean difference (MD) with a corresponding 95% confidence interval (CI), and dichotomous data were analyzed using the odds ratio (OR) with a corresponding 95% CI. A HR with 95% CIs was used for time-to-event outcomes (DFS and OS). Meta-analyses for these time-to-event data were conducted by pooling the log-transformed HR and its variance derived from each included study. Heterogeneity was evaluated based on the Q test and I^2^ statistics. The heterogeneity of effect sizes among the studies was assessed using the Q statistic (P < 0.05 was considered heterogeneous) and the I^2^ statistic (I^2^ >50% was regarded as heterogeneous). A random-effects model was used if significant heterogeneity was found among the studies; otherwise, a fixed-effects model was applied.

### Methods of evidence synthesis in NMA

The NMA was conducted within a Bayesian framework using JAGS version 4.3 to allow indirect comparisons among treatment interventions. The NMAs were fitted using Markov chain Monte Carlo techniques and implemented in JAGS. The analysis was performed using 1,000 burn-ins, 50,000 iterations, and 20,000 adaptations. Subsequently, the model fit was assessed using a leverage diagram. The fit of the random and fixed effect models was compared based on the deviance information criterion (DIC), with a lower DIC indicating superior model performance (31, 32). To evaluate inconsistency, the posterior mean deviance contributions of individual data points were plotted for the consistency model versus the inconsistency model, as suggested in NICE-DSU TSD 4 (31).

The NMA results were reported as median posterior MD (OR HR) with corresponding 95% credible intervals (CrIs). We used the surface under the cumulative ranking curve (SUCRA) and the mean ranks to evaluate the outcome of four surgical methods. For individual surgical methods, SUCRA values were computed for each outcome, ranging between 0 and 1, with values nearer to 1 indicating the preferred treatment. The sensitivity analyses were performed sequentially, excluding each incorporated study to discover significant changes in the combined results. Publication and reporting biases were assessed using adjusted funnel plots and the Egger test. All statistical analyses were conducted using “gemtc” and “BUGSnet” packages in R statistical software (version 3.6.2) and STATA (version 14.0) (33).

#### Subgroup analysis and investigation of heterogeneity

Previous studies have reported that the use of a uterine manipulator may impact DFS and OS (21, 34). Consequently, we reclassified the four surgical methods based on the use or non-use of a uterine manipulator for subgroup analysis. Additionally, we conducted subgroup analyses and meta-regression on outcomes with high heterogeneity in the PMA to investigate sources of heterogeneity.

## Results

### Literature search

**Figure 1:**
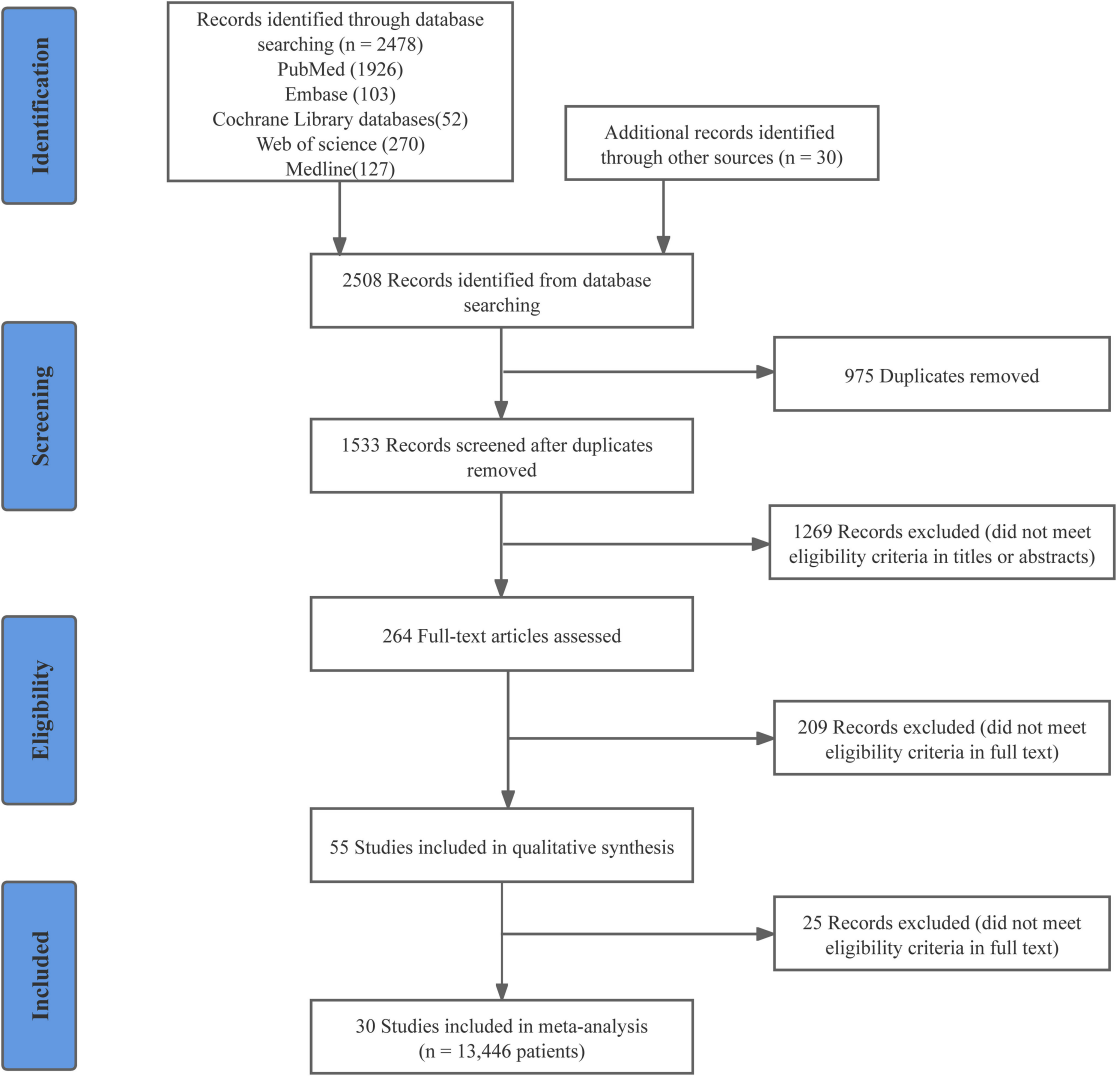
PRISMA flow diagram of study selection.

Following the previously established search strategy, 2508 potentially relevant articles were identified. After duplicate removal and initial screening, the number was reduced to 264. Subsequently, a full-text review was conducted to further refine the selection by excluding articles that did not meet the inclusion criteria, resulting in 55 studies being identified. Twenty-five observational studies were excluded for not meeting a NOS score 7 (**Supplementary Table 1**). Ultimately, 30 studies (18 RCTs and 12 observational studies) with 13446 patients were enrolled in this meta-analysis (35–64). Detailed information on the quality evaluation of RCTs and observational studies is displayed in **Supplementary Figure 1** and **Supplementary Table 2**. The PRISMA flow diagram visually represents the details of the article selection and exclusion procedure (**Figure 1**).

### Study characteristics

**Table 1** summarizes the characteristics of the included studies. The study period from 2001 to May 2017 involved EC patients at clinical stages I to IV. The mean age of the patients ranged from 49.88 to 69.30 years, and the mean body-mass index (BMI) varied from 23.38 to 34.20 kg/m². This analysis included 13,446 patients worldwide, with 5,730, 4,869, 924, and 1,923 patients undergoing OH, LH, RH, and LAVH, respectively. Among the 30 studies, 18 were RCTs, 5 were prospective studies, 3 were retrospective, 2 were prospective propensity score-matched, and 2 were retrospective propensity score-matched studies. Additionally, 16 studies were multicenter trials, while the other 14 were conducted at single centers. The majority of the studies originated from Europe (11/30, 37%) and America (10/30, 33%). The network relationships among surgical methods for each outcome are shown in **Supplementary Figure 2**.

**Table 1:** Characteristics of the 30 included studies.

Study	Year	Country	Type	Method	Age	BMI (kg/)	Sample size	Stage				Grade			Design	RER
								I	II	III	IV	1	2	3		
Kyrgiou et al.	2015	England et al.	MC	OH	63(57-70)	29(25-34)	1309	984	137	117	44	449	556	269	RCT	35
				LH	62(56-68)	30(25-35)	99	78	9	8	1	22	44	29		
Zullo et al.	2005	Italy	SC	OH	61.5±13.3	31.8±8.5	38	30	4	4	0	18	14	6	RCT	36
				LAVH	62.1±14.5	29.9±7.5	40	30	6	3	1	19	14	7
Lu et al.	2013	China	SC	OH	57.2(29-79)	27.1(17.8-40.0)	121	102	11	8	NA	80	31	10	RCT	37
				LH	56.6(27-82)	26.4(17.0-36.7)	151	137	10	4	NA	95	40	16
Janda et al.	2017	Australia et al.	MC	OH	63.1±10.6	32.7(19.1-63.2)	353	281	45	20	4	233	107	23	RCT	38
				LH	63.3±10.0	33.1(18.8-63.3)	407	341	32	27	3	259	120	28
Obermair et al.	2012	Australia et al.	MC	OH	32±9.2	NA	349	281	44	19	4	220	106	23	RCT	39
				LH	35±8.7	NA	404	340	33	26	3	258	119	27
Malzoni et al.	2009	Italy	SC	OH	63±14	29±7.3	78	66	5	7	NA	26	35	17	RCT	40
				LH	60±11	28±6.9	81	71	5	5	NA	24	40	17
Ghezzi et al.	2006	Italy	MC	LH	63.5±8.8	29.6±5.4	35	23	3	9	NA	11	19	5	RCT	41
				LAVH	63±8.9	27.9±5.4	37	31	1	5	NA	11	20	6
Malur et al.	2001	Germany	SC	OH	67.7(53-94)	NA	33	28	2	3	NA	15	11	7	RCT	42
				LAVH	68.3(48-85)	NA	37	31	3	3	NA	17	13	7
Zullo et al.	2009	Italy	MC	OH	NA	NA	38	30	4	4	4	18	14	6	RCT	43
				LAVH	NA	NA	40	30	6	3	1	19	14	7
FRAM et al.	2002	Australia	SC	OH	60.6	26.2	32	NA	NA	NA	NA	NA	NA	NA	RCT	44
				LAVH	61.2	25.7	29	NA	NA	NA	NA	NA	NA	NA
Walker et al.	2009	American et al.	MC	OH	63(55-71)	29(24-34)	886	680	52	126	28	NA	NA	NA	RCT	45
				LAVH	63(55-72)	28(24-34)	1630	1253	98	239	39	NA	NA	NA
Tozzi et al.	2005	Germany	SC	OH	66(36-89)	NA	59	54	3	2	NA	21	25	13	RCT	46
				LAVH	67(35-88)	NA	63	64	5	4	NA	20	26	17
Somashekhar et al.	2014	India	SC	OH	53	31.88	25	NA	NA	NA	NA	NA	NA	NA	RCT	47
				RH	51.44	30.96	25	NA	NA	NA	NA	NA	NA	NA
Mourits et al.	2010	Netherlands	MC	OH	63(39-86)	28(19-48)	94	75	8	2	1	55	26	5	RCT	48
				LH	62(40-89)	29(17-55)	185	130	15	2	2	107	32	10
Janda et al.	2010	Australia et al.	MC	OH	62.7±9.7	NA	142	117	16	7	1	89	46	7	RCT	49
				LH	62.8±10.0	NA	190	163	14	11	0	122	59	9
Kornblith et al.	2009	American et al.	MC	OH	62.5±11.8	NA	267	NA	NA	NA	NA	NA	NA	NA	RCT	50
				LH	64.1±11.2	NA	535	NA	NA	NA	NA	NA	NA	NA
Walker et al.	2012	American et al.	MC	OH	62.7(54.9-70.6)	28.5(24.2-34.2)	920	714	34	105	28	NA	NA	NA	RCT	51
				LH	62.8(55.4-71.6)	28.4(24.4-34.0)	1696	1332	65	185	39	NA	NA	NA
Zorlu et al.	2005	Turkey	SC	OH	54.9(36-77)	26.2	26	14	4	8	NA	14	6	6	RCT	52
				LH	56.6(40-72)	24.4	26	21	2	3	NA	15	5	6
Lim et al.	2011	American	MC	LH	61.6±11.8	29.9±7.0	122	NA	NA	NA	NA	NA	NA	NA	Pro Ma	53
				RH	62.1±8.4	31.0±8.8	122	NA	NA	NA	NA	NA	NA	NA
Eklind et al.	2015	Sweden	MC	OH	66(44-84)	29(19-44)	48	NA	NA	NA	NA	NA	NA	NA	Pro	54
				RH	66(47-87)	29(19-46)	40	NA	NA	NA	NA	NA	NA	NA
Limet al.	2010	American	SC	OH	62.7±10.6	28.9±9.4	36	NA	NA	NA	NA	NA	NA	NA	Pro Ma	55
				LH	61.4±11.7	28.2±6.7	56	NA	NA	NA	NA	NA	NA	NA
				RH	62.5±8.4	30. ±8.8	56	NA	NA	NA	NA	NA	NA	NA	
Seamon et al.	2009	American	MC	LH	57±11	28.7±6.9	76	86	5	NA	NA	77	12	11	Pro	56
				RH	59±8.9	34.2±9	105	87	3	NA	NA	66	22	12
JUNG et al.	2010	Korea	SC	OH	50.20±8.06	24.82±4.03	56	NA	NA	NA	NA	NA	NA	NA	Pro	57
				LH	49.88±10.75	25.17±5.11	25	NA	NA	NA	NA	NA	NA	NA
				RH	52.89±11.91	23.38±3.08	28	NA	NA	NA	NA	NA	NA	NA	
Estape et al.	2012	American	MC	OH	64.9±12.2	33.1±8.2	78	51	7	16	4	15	30	30	Pro	58
				LH	60.8±13.2	30.3±6.9	104	94	5	5	0	36	51	17
				RH	64.0±14.5	31.5±8.3	102	90	6	3	2	24	52	26	
Veljovich et al.	2008	American	MC	OH	63(30–92)	32.2 (16.4-65.8)	131	NA	NA	NA	NA	NA	NA	NA	Pro	59
				RH	59.5 (5)	27.6(18.7-49.4)	25	NA	NA	NA	NA	NA	NA	NA
Manchana et al.	2015	Thailand	SC	OH	59(53-65)	25.4(22.5-30.2)	143	94	12	27	10	76	26	41	Retro	60
				LH	54(49-62)	24.5(21.8-28.3)	47	42	1	4	0	35	7	5
				RH	55.5(48.2-61.5)	26.8(22.7-35.6)	28	20	6	2	0	20	3	5	
Coronado et al.	2012	Spain	SC	OH	64.7±11.2	29.5±6.6	192	151	5	30	6	112	40	40	Retro	61
				LH	65.9±11.2	27.2±5.3	84	72	4	6	2	51	20	13
				RH	67.3±10.2	28.7±4.7	71	57	4	9	1	45	12	14	
Corrado et al.	2015	Italy	SC	OH	63(38-88)	28(17-80)	177	139	12	20	6	31	93	53	Retro Ma	62
				LH	62(28-86)	29(17-59)	277	237	15	22	3	59	150	68
				RH	64(35-90)	29(20-42)	72	57	8	6	1	10	38	24	
Goicoechea et al.	2013	American	MC	LH	61(27-86)	29.3(17-58)	232	197	12	21	2	113	72	47	Retro	63
				RH	62(39-86)	29.2(17-55)	183	153	4	23	3	79	52	52
Magrina et al.	2011	American	SC	OH	65.2±11.4	30.5±9.1	99	65	2	17	15	59	150	68	Retro Ma	64
				LH	69.3±9.4	27.3±7.6	37	27	3	4	3	10	38	24
				RH	64.6±11.9	30.7±10.0	67	55	5	7	0	113	72	47	
				LAVH	68.5±10.9	26.9±5.0	47	40	1	5	1	79	52	52	

BMI, body-mass index; MC, Multicenter; SC, Single center; OH, open hysterectomy; LH, laparoscopic hysterectomy; RH, robotic hysterectomy; LAVH, laparoscopic-assisted vaginal hysterectomy; NA, not available; Retro, retrospective study; Retro Ma, retrospective propensity score-matched study; Pro, prospective study; Pro Ma, prospective propensity score-matched study; RER, references.

### Surgical efficacy

#### DFS and OS

**Figure 2:**
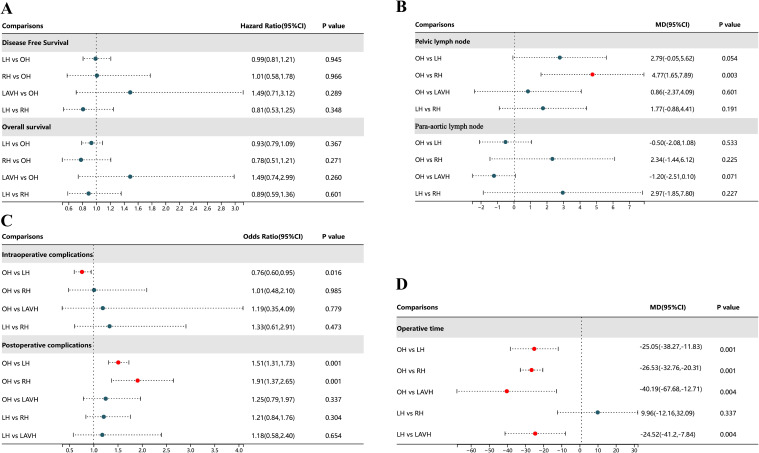
Forest plot comparison of the different surgical methods for all outcomes. OH, open hysterectomy; LH, laparoscopic hysterectomy; RH, robotic hysterectomy; LAVH, laparoscopic-assisted vaginal hysterectomy; HR, hazard ratio; OR, odds ratio; 95% CI, 95% credibility intervals; MD, mean difference.

**Figure 3:**
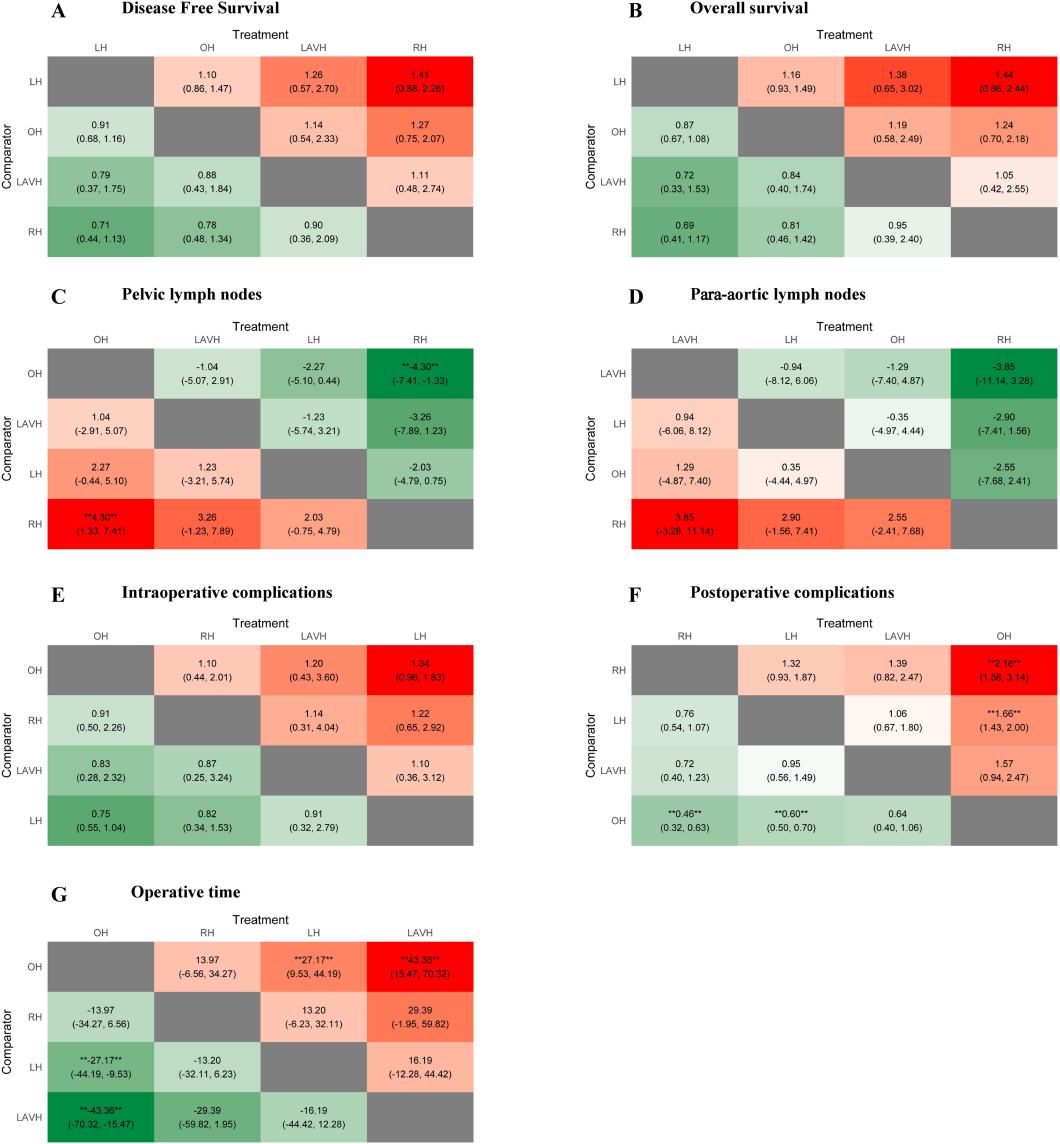
Heat plots of the league table for the four surgical methods. OH, open hysterectomy; LH, laparoscopic hysterectomy; RH, robotic hysterectomy; LAVH, laparoscopic-assisted vaginal hysterectomy.

**Figure 4:**
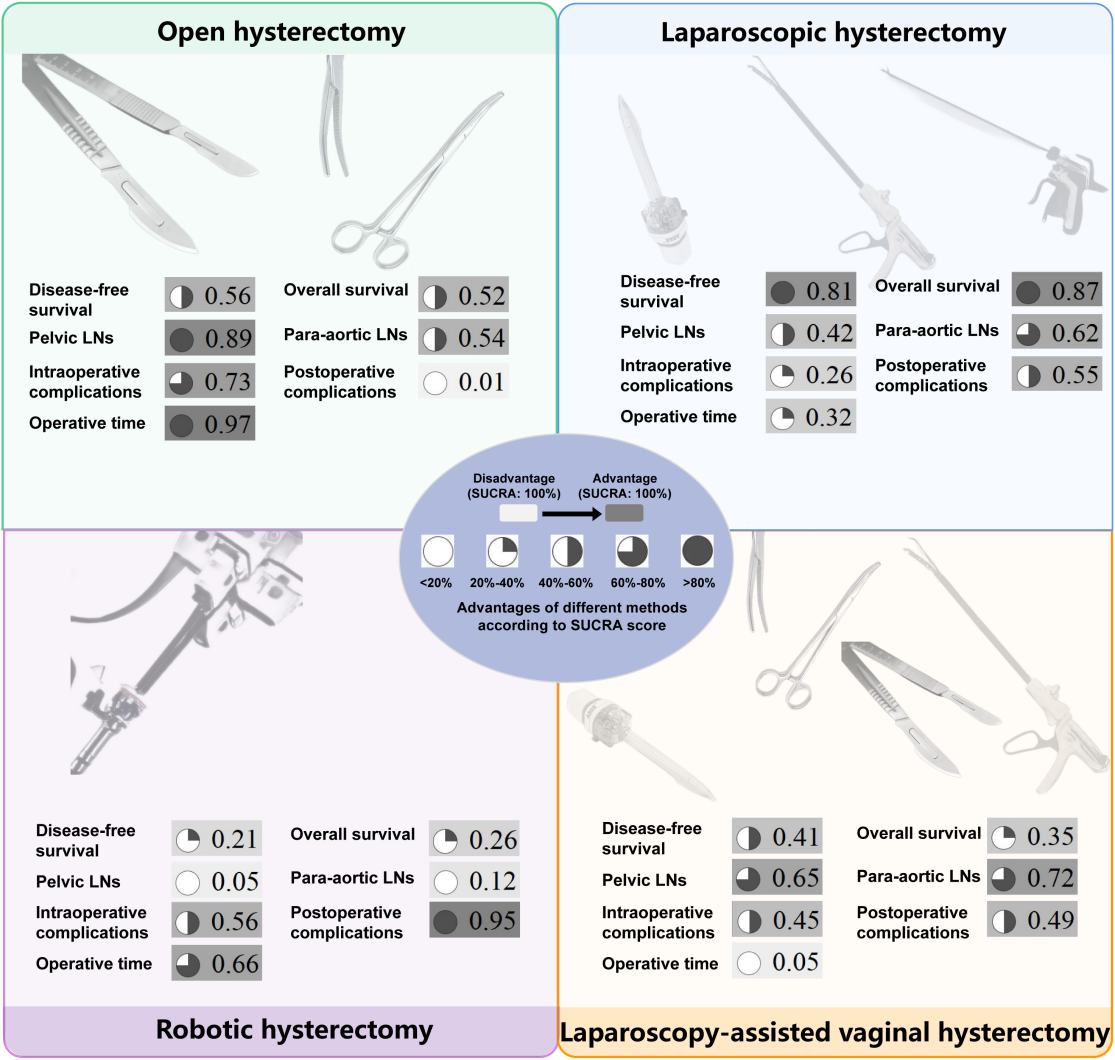
The surface under the cumulative ranking curve values of all outcomes for four surgical methods. The darker color, consistent with the higher SUCRA value, indicates greater superiority. SUCRA, surface under the cumulative ranking curve; LNs, lymph nodes.

There were no statistically significant differences in DFS and OS among the four surgical methods in both PMA and NMA (**Figures 2A**, **3A, B**). The rankings of four surgical methods from worst to best (1st to 4th) for DFS and OS are presented in **Supplementary Figure 3** and **Supplementary Table 3**. The SUCRA values of DFS in OH, LH, RH and LAVH were 0.56, 0.81, 0.21 and 0.41, respectively. For OS, the SUCRA values of OH, LH, RH and LAVH were 0.52, 0.87, 0.26 and 0.35, respectively. Notably, LH exhibited the highest performance in both DFS and OS, with SUCRA values of 0.81 and 0.87, respectively. In contrast, RH showed the least advantage in both DFS and OS, with SUCRA values of 0.21 and 0.26, respectively (**Figure 4**).

#### Number of retrieved lymph nodes

Compared to RH, OH was associated with a higher number of retrieved pelvic lymph nodes in both PMA (MD: 4.77, 95% CI: 1.65-7.89, p = 0.003) and NMA (MD: 4.30, 95% CI: 1.33-7.41) (**Figures 2B**, **3C**). Nonetheless, PMA and NMA indicated no significant statistical differences in the para-aortic lymph node retrieval across all four surgical methods (**Figures 2B**, **3D**). Overall, OH had the highest SUCRA value for the number of retrieved pelvic lymph nodes (0.89). LAVH demonstrated the most effective capability in the para-aortic lymph node dissection, with a SUCRA value of 0.72 (**Figure 4**). More results involving the SUCRA values and rankings of other surgical methods for retrieved lymph nodes are shown in **Supplementary Figure 3** and **Supplementary Table 3**.

#### Surgical safety

In the PMA, OH demonstrated a reduced intraoperative complication rate compared to LH (OR: 0.76, 95% CI: 0.60-0.95, P = 0.016) (**Figure 2C**). However, the NMA revealed no significant differences in intraoperative complication rates among four surgical methods (**Figure 3E**). Both the PMA (OR: 1.91, 95% CI: 1.37-2.65, P = 0.001) and NMA (OR: 2.16, 95% CI: 1.58-3.14) disclosed that OH had a higher postoperative complication rate than RH (**Figure 2C** and **Figure 3F**). Furthermore, OH may increase the postoperative complication rate compared to LH in the PMA (OR: 1.51, 95% CI: 1.31-1.73, P = 0.001) (**Figure 2C**). Notably, RH showed the most significant benefits in minimizing postoperative complications, evidenced by the highest SUCRA values of 0.95. In comparison, OH had the most unfavorable postoperative outcomes, with a SUCRA value of only 0.01. However, OH achieved the highest SUCRA value (0.73) for intraoperative complications (**Figure 4**). More results regarding the SUCRA values of other surgical methods for surgical safety are available in **Supplementary Table 3**.

#### Operative time

Notable differences in operative times were observed in four surgical methods. Both PMA and NMA indicated LH and LAVH required more operative time than OH (**Figures 2D**, **3G**). RH was associated with a longer operative time relative to OH in PMA (MD: 26.53, 95% CI: 20.31-32.76, p = 0.001) (**Figure 2D**). In addition, LAVH also had a longer operative time comparison to LH shown in PMA (MD: 24.52, 95% CI: 7.84-41.2, p = 0.004) (**Figure 2D**). Generally, OH significantly reduced operative time, surpassing all other surgical methods, as evidenced by the highest SUCRA value of 0.97 (**Figure 4**).

### Inconsistency, sensitivity analysis, and publication bias

The leverage plots with DIC values are shown in **Supplementary Figure 4**. There was a lack of evidence to suggest inconsistency within this NMA for four surgical methods, as shown in **Supplementary Figure 5**. We conducted a sensitivity analysis by removing individual studies and modifying the effect models. The overall statistical significance did not change, suggesting the robustness and reliability of our findings. The publication biases of these 30 included studies were assessed by funnel plots. The shape of funnel plots showed no obvious asymmetry except for operative time and number of retrieved pelvic lymph nodes (**Supplementary Figure 6**). Subsequently, the Egger test showed no significant P values for operative time (P = 0.61) or pelvic lymph nodes (P = 0.89) (**Supplementary Table 4**).

### Subgroup analysis and meta-regression analysis

After consulting the authors via email or conducting a thorough review of the full texts, we ultimately identified 8 studies out of 30, reclassifying the surgical methods into OH, LH-U (use of uterine manipulator), LH-NU (non-use of uterine manipulator), LAVH-U (use of uterine manipulator), and LAVH-NU (non-use of uterine manipulator). RH was excluded due to the absence of applicable data. In the NMA, no statistically significant differences were observed for DFS in five subgroups (**Supplementary Figure 7**). The SUCRA values of DFS in OH, LH-U, LH-NU, LAVH-U and LH-NU were 0.45, 0.34, 0.62, 0.52, and 0.55, respectively. SUCRA values in LH and LAVH groups without uterine manipulators were higher than in those with manipulators. Besides, OS was not further analyzed due to insufficient data (**Supplementary Table 5**). We performed Meta-regression analyses of operative time and pelvic lymph node to investigate possible heterogeneity. The analysis revealed that publication years, types, or countries had no significant impact on operative time or pelvic lymph nodes (**Supplementary Table 6**).

## Discussion

Traditional meta-analyses can only perform pairwise comparisons between different treatments and cannot comprehensively compare three or more treatments. However, NMA is able to quantitatively assess three or more treatments by calculating SUCRA values (33). This study is the first NMA to compare the surgical efficiency and safety of four kinds of hysterectomy for EC patients. To enhance the reliability of the result, we included only RCTs for OH, LH, and LAVH. For RH, we incorporated high-quality observational studies. Additionally, LH and LAVH were further subdivided into LH-U, LH-NU, LAVH-U, and LAVH-NU based on the use of a uterine manipulator. This study is the first to simultaneously discuss the impact of different surgical methods and the use of a uterine manipulator on DFS separately.

As the most important prognostic indicators for patients, DFS and OS also reflect surgical efficacy. Our study found that there were no significant differences in DFS and OS among the four surgical methods. According to SUCRA values, LH ranked the highest in DFS and OS. Conversely, RH ranked the lowest, indicating that RH may have a relative disadvantage in surgical efficacy. Notably, there was a controversy regarding the impact of uterine manipulators on surgical efficacy. A multicenter retrospective study by Padilla-Iserte et al. demonstrated a significant association between the use of uterine manipulators and an increased risk of recurrence (20). Additionally, the use of uterine manipulators in uterus-confined EC (International Federation of Gynecology and Obstetrics [FIGO] Stage IeII) was connected to lower disease-free survival (20). On the contrary, a recent meta-analysis demonstrated that the use of uterine manipulators during hysterectomy for EC was not significantly associated with recurrence-free or overall survival (21). However, they did not rule out the influence of different surgical methods on oncological outcomes. To address this, we conducted subgroup analyses by subdividing LH into LH-NU and LH-U, and RH into RH-NU and RH-U. The results showed that the use of uterine manipulators did not have a significant effect on DFS. Notably, based on SUCRA values, we found that LH and RH performed without the use of uterine manipulators had a more advantage compared to those performed with uterine manipulators. This suggests that there may be a potential impact of uterine manipulators on DFS. Further large-scale, multicenter RCTs are needed to explore the influence of uterine manipulators on surgical efficacy within the same surgical method.

In the absence of sentinel lymph node techniques, the number of retrieved lymph nodes can reflect the staging efficacy of different surgical methods for EC. During the surgery, the lymph nodes that need to be dissected primarily include pelvic and para-aortic lymph nodes. The results showed that the number of retrieved pelvic lymph nodes in RH is significantly lower than that in OH. Furthermore, based on the SUCRA values, RH has the least advantage in terms of the number of lymph nodes dissected, suggesting that RH may have certain limitations in lymph node dissection. Notably, we also found that in other types of cancers, robotic surgery similarly exhibited certain disadvantages in lymph node dissection (65–67). The reason might be the lack of tactile feedback, which can undermine the surgeon’s confidence during lymph node dissection. Surgeons may be concerned about damaging surrounding critical organs such as arteries and ureters. Therefore, finding ways to enhance the capability of lymph node dissection for RH is a promising direction in the future. Therefore, RH may not be suitable for EC patients with preoperative computed tomography indicating suspicious lymph node metastasis. On the contrary, OH showed the best advantage in dissecting pelvic lymph nodes. Therefore, OH is ideal for patients with suspicious pelvic lymph node metastasis. The surgical procedure for para-aortic lymph node dissection in LAVH and LH are the same. Our results also showed that LAVH and LH have similar advantages in dissecting para-aortic lymph nodes (SUCRA values 0.72 and 0.62). Therefore, both of them may be ideal for patients with suspicious para-aortic lymph node metastasis.

The intraoperative complications mainly include hemorrhage, bladder injury, ureteral injury, nerve injury, and bowel injury. The findings of this study align with previous research, showing that OH has an advantage in reducing intraoperative complications compared to LH and RH (23, 68). This is likely due to the extensive experience of surgeons and the clear and direct surgical field. Additionally, OH allows for more flexible and versatile maneuvers, such as using a finger to palpate arterial pulses to avoid major hemorrhage effectively. LH has the lowest SUCRA values, possibly due to the limitations imposed by instrument manipulation during surgery. Notably, some of the RCTs included in our study were conducted in earlier years, and the learning curve may have contributed to an increased incidence of intraoperative complications in LH (45, 49). The expertise level of surgeons is also a significant factor, but the studies included in our analysis rarely reported specific details such as the number of procedures completed, which prevented us from exploring this topic further. RH ranks second in SUCRA values, likely because the 3D visual field and articulated arms of RH enable precise and flexible operations. The postoperative complications mainly include infection, venous thromboembolism, bladder dysfunction, bowel obstruction, and lymphedema. The highest incidence of postoperative complications in OH may be related to the long abdominal incision, significant trauma, and prolonged recovery time (6, 69). Similar to previous research findings, RH has the most advantage in reducing postoperative complications (12, 23, 70). This may be due to the small surgical incision, precise operation, and relatively minimal tissue damage associated with RH. This advantage is consistent with surgical outcomes in other malignancies (65, 71, 72). Although RH is inferior to other surgical methods in terms of DFS, OS, and the number of lymph nodes dissected, its surgical safety is the best.

Operative time is a crucial measure of surgical efficiency. Our study revealed that LAVH had the longest operative time. This was likely due to the complexity of LAVH, which required both laparoscopic and vaginal steps (41). Moreover, the confined space during the vaginal step limited operation, contributing to the extended operative time (44, 73). By contrast, OH had a significantly shorter operative time compared to LH, LAVH, and RH, consistent with previous researches (68, 74, 75). Therefore, OH is a reasonable option for EC patients with high anesthesia risks or those needing shorter surgical duration. It is worth noting that some of the studies included in this analysis are relatively early. As surgical skills improve, the operative time difference between OH and other surgical methods is expected to decrease. Although our study focused on four common surgical methods, we acknowledge the growing interest in Vaginal Natural Orifice Transluminal Endoscopic Surgery (V-NOTES) as an emerging minimally invasive approach for hysterectomy. V-NOTES improved cosmetics without scars on the abdomen and reduced recovery time. V-NOTES hysterectomy has a steep learning curve and is technically challenging, requiring a high level of surgical expertise. Recent research suggests that V-NOTES hysterectomy can achieve satisfactory outcomes for benign diseases by experienced surgeons (76). However, only a few studies have reported its application in the treatment of EC (77, 78). Future large cohort RCTs are needed to evaluate the safety and long-term oncological outcomes of V-NOTES hysterectomy for EC.

This study has some limitations. Firstly, due to the relatively small number of RCTs available for RH, we included some high-quality observational studies. Secondly, we were unable to analyze any single specific complication (such as intestinal obstruction) due to the types of surgical complications varying among different original studies. More studies are needed in the future to investigate this issue further. Thirdly, we cannot analyze the OS of the five subgroups because of insufficient data.

## Conclusion

The benefits and limitations of each surgical method for EC are obvious according to SUCRA values. There are no significant differences among the four surgical methods in DFS or OS. The use of uterine manipulators does not affect prognosis. OH is the best method for shortening operative time, dissecting the pelvic lymph nodes and controlling intraoperative complications while having disadvantages in postoperative complications. LH has advantages in DFS, OS, and para-aortic lymph node dissection but has limitations in intraoperative complications. LAVH also has an advantage in para-aortic lymph node dissection while having the longest operative time. RH has advantages in controlling surgical complications while having some limitations in DFS, OS and lymph node dissection.

## Data Availability

The original contributions presented in the study are included in the article/**Supplementary Material**. Further inquiries can be directed to the corresponding authors.
