# Supplementary material for: Different surgical methods of hysterectomy for the management of endometrial cancer: a systematic review and network meta-analysis
Source: Front Oncol. 2025 Jan 15;14:1524991. doi: 10.3389/fonc.2024.1524991 (PMC11774694; doi:10.3389/fonc.2024.1524991)
Supplement: Supplementary file 1 [file DataSheet1.pdf]

Supplementary search terms

Pubmed:

((((("Endometrial Neoplasms"[Mesh]) OR (Endometrial Neoplasm[Title/Abstract])) OR (Neoplasm, Endometrial[Title/Abstract])) OR (Neoplasms, Endometrial[Title/Abstract])) OR (Endometrial Carcinoma[Title/Abstract])) OR (Carcinoma, Endometrial[Title/Abstract])

AND/OR

((((("Hysterectomy"[Mesh]) OR (Hysterectomies[Title/Abstract])) OR (Abdominal Hysterectomy[Title/Abstract])) OR (laparotomy[Title/Abstract])) OR (conventional Hysterectomy[Title/Abstract])) OR (Open Hysterectomy[Title/Abstract])

AND/OR

((((("Hysterectomy, Vaginal"[Mesh]) OR (laparoscopic-assisted vaginal hysterectomy[Title/Abstract])) OR (Laparoscopically assisted vaginal hysterectomy[Title/Abstract])) OR (Hysterectomies, Vaginal[Title/Abstract])) OR (Vaginal Hysterectomies[Title/Abstract])) OR (Vaginal Hysterectomy[Title/Abstract])

AND/OR

(((("Endoscopy"[Mesh]) OR (laparoscopy [Title/Abstract])) OR (endoscopy[Title/Abstract])) OR (minimally invasive surgery[Title/Abstract])) OR (video-assisted surgery[Title/Abstract])

AND/OR

((((("Robotic Surgical Procedures"[Mesh]) OR (robotic hysterectomy [Title/Abstract])) OR (da Vinci surgical system[Title/Abstract])) OR (Robotic Surgical Procedure[Title/Abstract])) OR (Surgical Procedure[Title/Abstract])) OR (Robot-Assisted hysterectomy [Title/Abstract])

Web of science:

ALL = (Endometrial Cancer* OR Endometrial Neoplasm* OR Endometrial Carcinoma)

AND/OR ALL = (Hysterectomy* OR laparotomy OR Abdominal Hysterectomy OR Open Hysterectomy)

AND/OR ALL = (Vaginal Hysterectomy* OR laparoscopic-assisted vaginal hysterectomy OR Laparoscopically assisted vaginal hysterectomy)

AND/OR ALL = (Endoscopy* OR laparoscopy* OR minimally invasive surgery OR video-assisted surgery)

AND/OR ALL = (Robotic Surgical Procedure* OR robotic hysterectomy* OR da Vinci surgical system OR Robot-Assisted hysterectomy)

Embase:

Endometrial Cancer*':ti AND/OR laparoscopic-assisted vaginal hysterectomy:ti AND/OR minimally invasive surgery:ti AND/OR Robotic hysterectomy:ti

Medline:

#1((AB=(Endometrial Neoplasms)) OR AB=(Endometrial Carcinoma)) OR AB=(Endometrial Cancer)

#2 ((AB=(Hysterectomy)) OR AB=(Abdominal Hysterectomy)) OR AB=(Open Hysterectomy)

#3 ((AB=(laparoscopy)) OR AB=(minimally invasive surgery)) OR AB=(video-assisted surgery)

#4 ((AB=(laparoscopic-assisted vaginal hysterectomy)) OR AB=(Laparoscopically assisted vaginal

hysterectomy)) OR AB=(Vaginal Hysterectomy)
#5 (AB=(robotic hysterectomy)) OR AB=(Robot-Assisted hysterectomy)
#6 #1 AND/OR #2 AND/OR #3 AND/OR #4 AND/OR #5

Cochrane Library databases:
kw'"endometrial cancer":t.ab,kw
AND/OR ("abdominal hysterectomy"):ti ab,.kw
AND/OR ("robotassisted aparoscopic surgeries"):ti.ab .kw
AND/OR ("laparoscopicaly assisted vaginal hysterectomy"):ti,ab,kw
AND/OR ("minimally-invasive surgery"):ti,ab,kw

Supplementary Figure 1

	Random sequence generation (selection bias)	Allocation concealment (selection bias)	Blinding of participants and personnel (performance bias)	Blinding of outcome assessment (detection bias)	Incomplete outcome data (attrition bias)	Selective reporting (reporting bias)	Other bias
Fram 2002	?	?	?	?	?	?	?
Ghezzi 2006	?	?	?	?	+	?	?
Janda 2010	+	+	?	?	+	?	?
Janda 2017	+	+	?	?	+	?	?
Kornblith 2009	?	?	?	?	+	?	?
Kyrgiou 2015	+	+	?	?	+	+	?
Lu 2013	+	+	?	?	+	?	?
Malur 2001	?	?	?	?	?	?	?
Malzoni 2009	+	+	?	?	+	?	?
Mourits 2010	+	+	?	?	+	?	?
Obermair 2012	+	+	?	+	?	+	?
Somashekhar 2014	?	−	?	?	?	?	?
Tozzi 2005	+	+	?	?	?	−	?
Walker 2009	+	+	?	?	+	?	?
Walker 2012	+	+	?	?	+	+	?
Zorlu 2005	?	?	?	?	+	?	?
Zullo 2005	+	+	?	?	+	?	?
Zullo 2009	+	+	?	?	+	+	?

Supplementary Figure 2

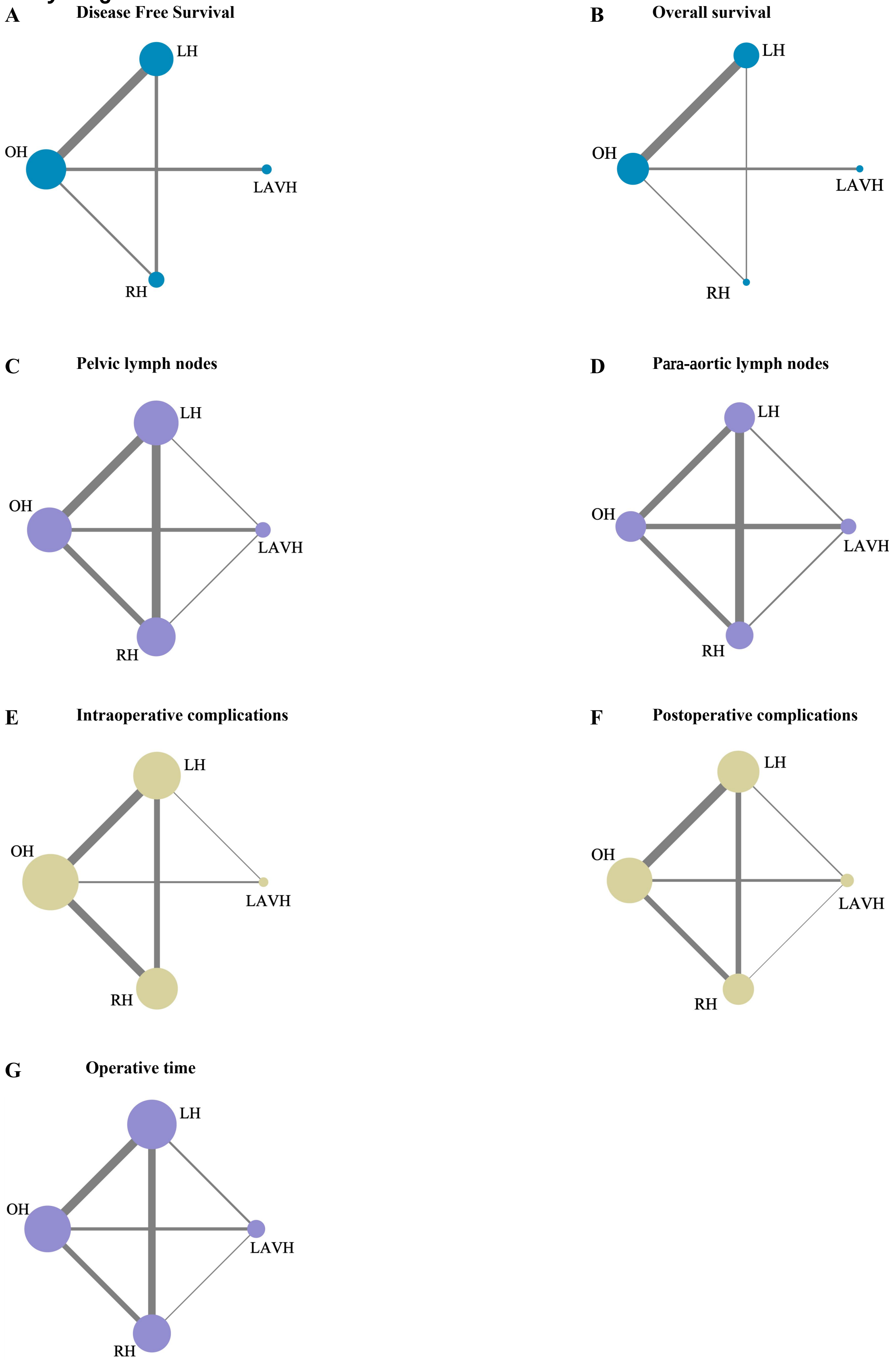

Supplementary Figure 3

A Disease Free Survival

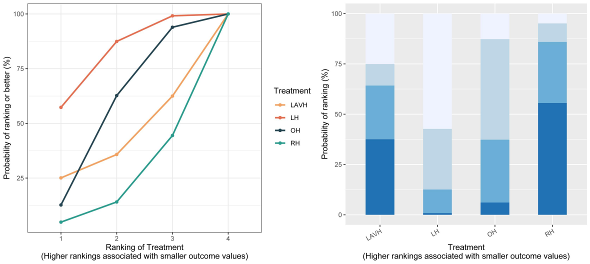

B Overall survival

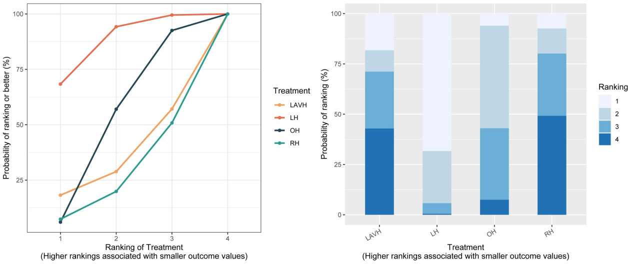

C Pelvic lymph nodes

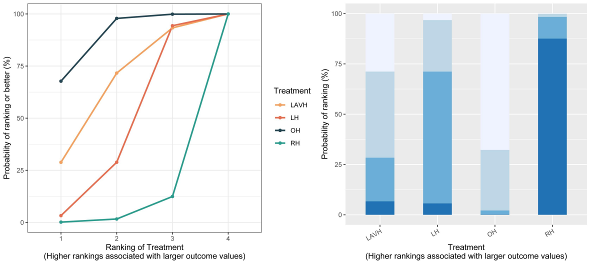

D Para-aorticlymph nodes

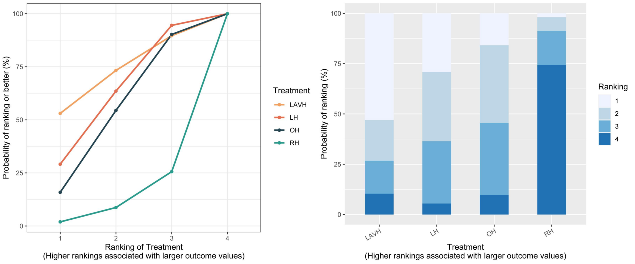

E Intraoperative complications

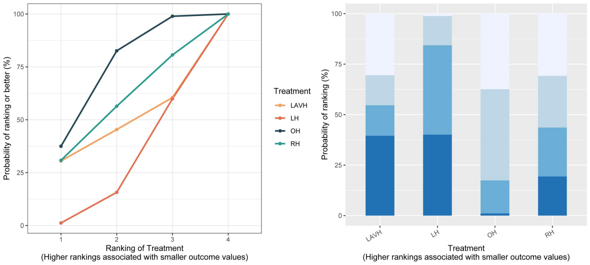

F Postoperative complications

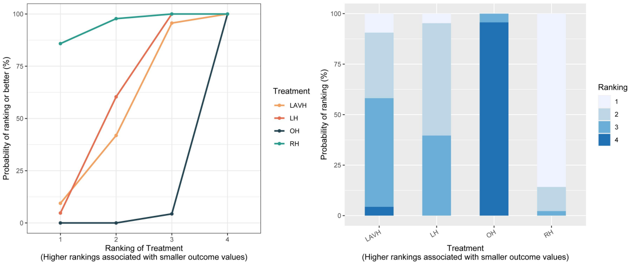

G Operative time

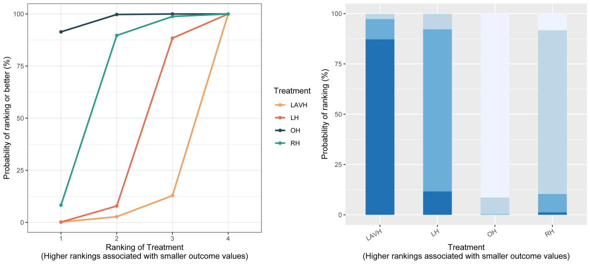

Supplementary Figure 4

A Disease Free Survival

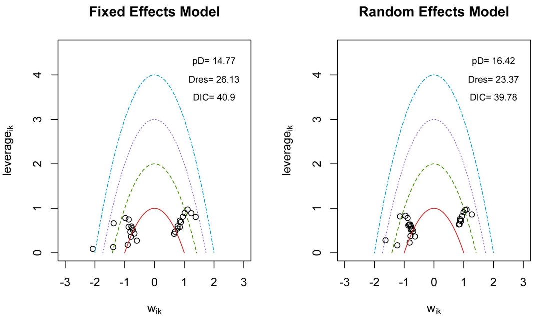

B Overall survival

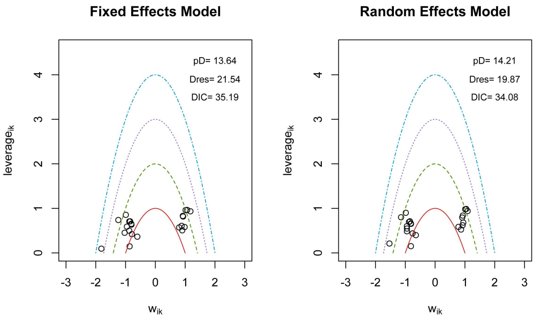

C Pelvic lymph nodes

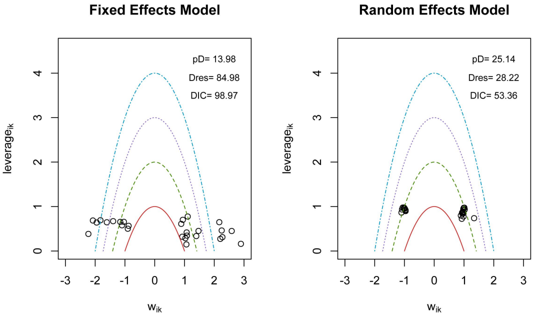

D Para-aortic lymph nodes

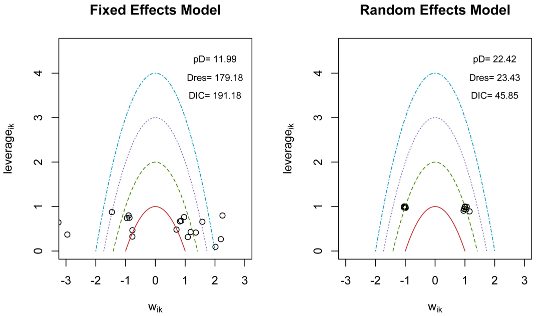

E Intraoperative complications

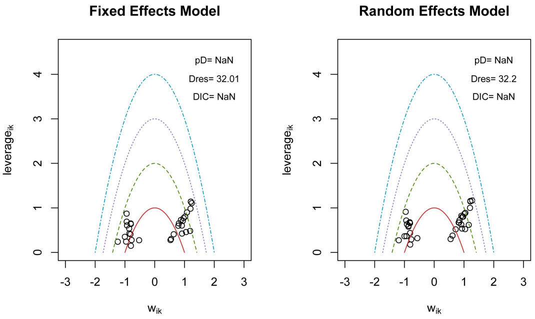

F Postoperative complications

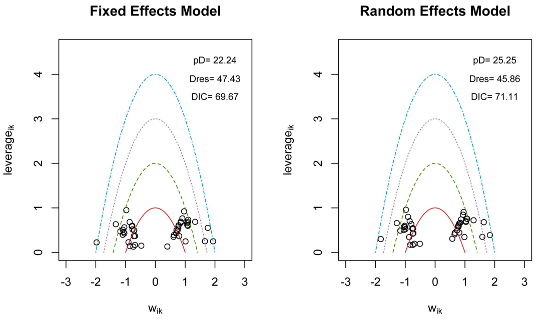

G Operative time

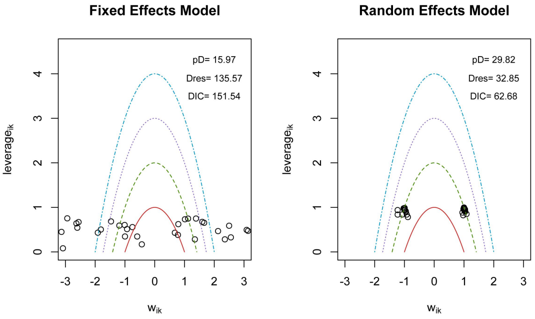

Supplementary Figure 5

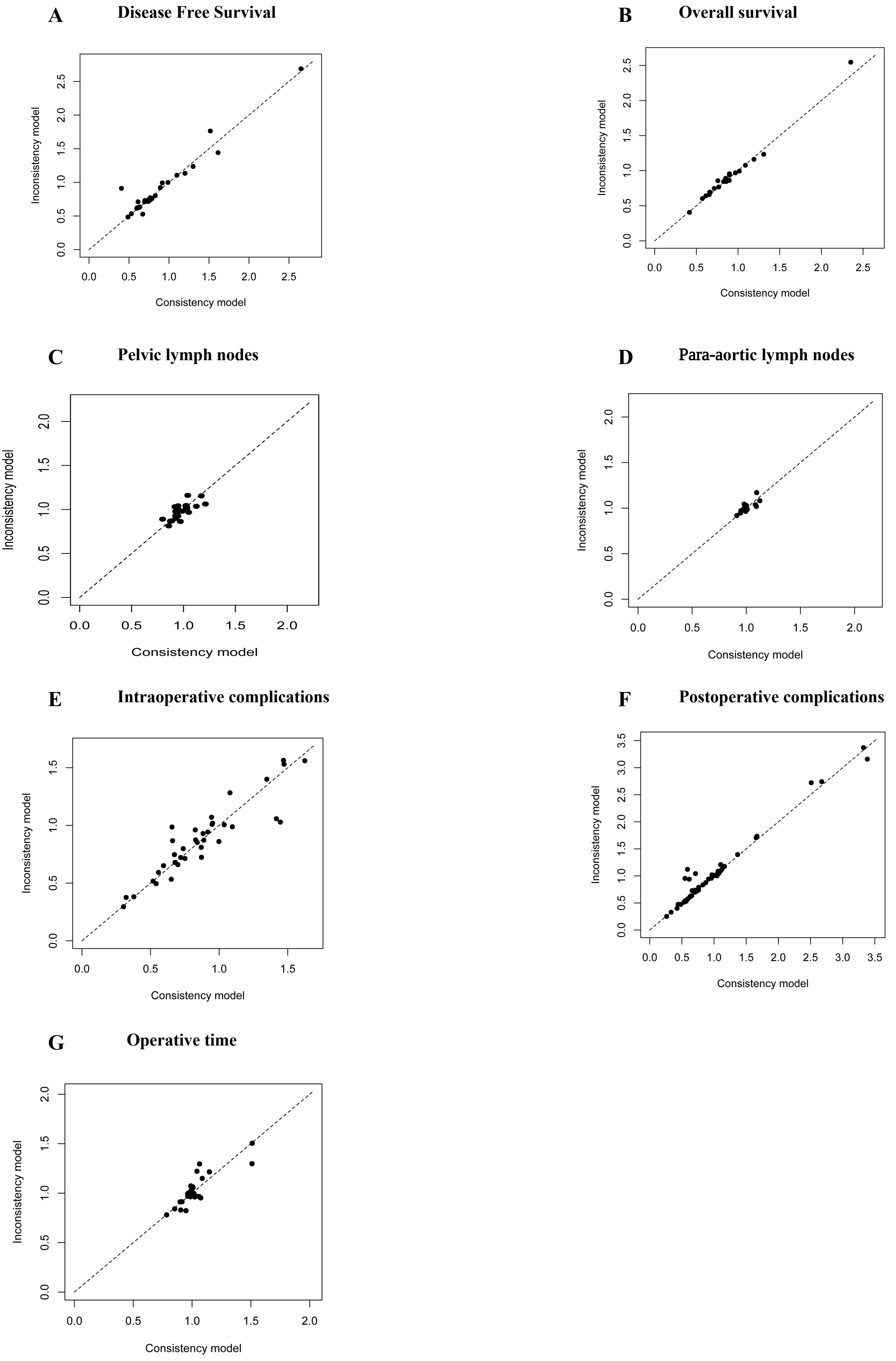

Supplementary Figure 6

A Disease Free Survival

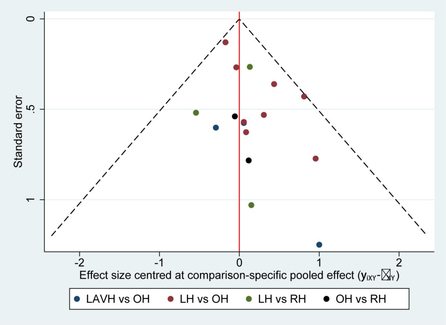

B Overall survival

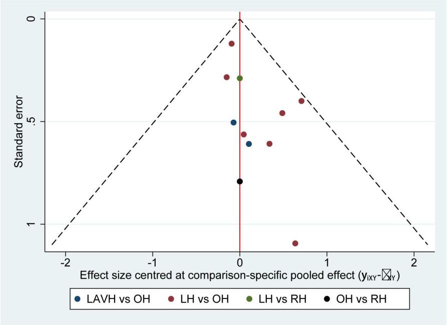

C Pelvic lymph nodes

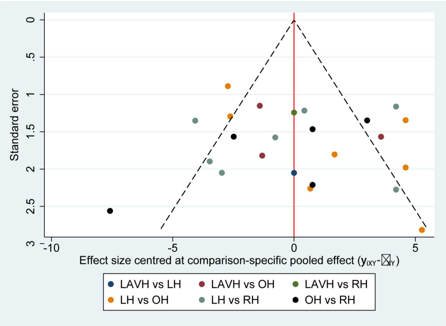

D Para-aortic lymph nodes

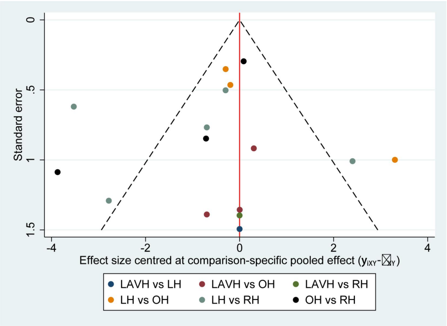

E Intraoperative complications

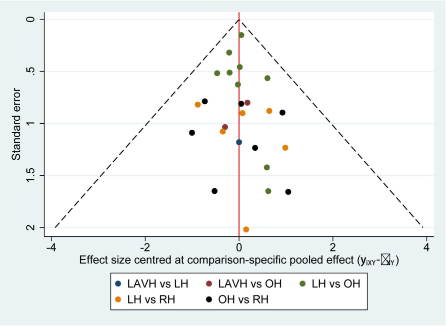

F Postoperative complications

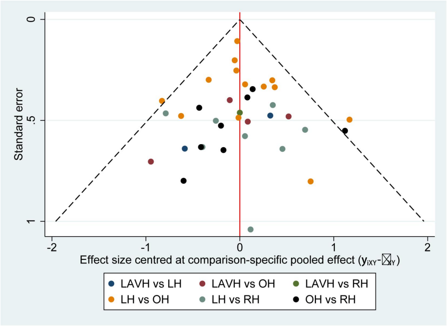

G Operative time

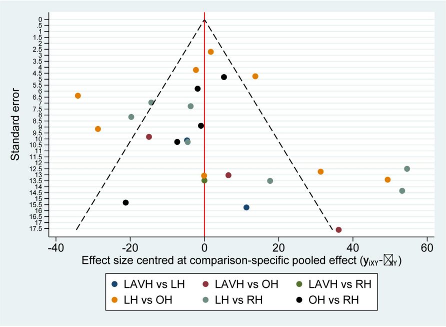

Supplementary Figure 7

Treatment (columns) vs. Comparator (rows)

Comparator \ Treatment	LH-NU	LAVH-NU	LAVH-U	OH	LH-U
LH-NU		1.10 (0.27, 4.33)	1.18 (0.24, 4.83)	1.25 (0.47, 3.54)	1.31 (0.48, 3.84)
LAVH-NU	0.91 (0.23, 3.69)		1.05 (0.24, 3.99)	1.14 (0.44, 3.08)	1.18 (0.45, 3.32)
LAVH-U	0.85 (0.21, 4.17)	0.95 (0.25, 4.09)		1.11 (0.46, 3.02)	1.17 (0.47, 3.15)
OH	0.80 (0.28, 2.11)	0.88 (0.32, 2.30)	0.90 (0.33, 2.17)		1.05 (0.85, 1.31)
LH-U	0.76 (0.26, 2.08)	0.84 (0.30, 2.24)	0.86 (0.32, 2.13)	0.96 (0.77, 1.18)	

Supplementary Table1. The quality of excluded observational study assessed by the Newcastle-Ottawa scale

Study	Selection				Comparability	Outcome			Quality score
	Representativeness of the exposed cohort	Selection of the non-exposed cohort	Ascertainment of exposure	Demonstration that outcome of interest was not present at start of study	Comparability of cohorts on the basis of the design or analysis	Assessment of outcome	Was follow-up long enough for outcomes to occur	Adequacy of follow up of cohorts	
Subramaniam et al.	★	★	★	★	★				5
Boggess et al.	★	★	★	★					4
Martino et al.	★	★	★	★	★	★			6
Venkat et al.	★	★	★	★					4
Johnson et al.	★	★	★	★					4
Mok et al.	★	★	★	★					4
ElSahwi et al.	★	★	★	★	★				5
Bell et al.	★	★	★	★	★	★			6
Nevadunsky et al.	★	★	★	★	★				5
Göçmen et al.	★	★	★	★	★				5
Seamon et al.	★	★	★	★					4

Mendivil et al.	★	★	★	★		★	5
Holtz et al.	★	★	★	★			4
Lavoue et al.	★	★	★	★	★	★	6
Goicoechea et al.	★	★	★	★	★		5
Hoekstra et al.	★	★	★	★		★	5
Soto et al.	★	★	★	★	★		5
Gehrig et al.	★	★	★	★			4
Turunen et al.	★	★	★	★	★		5
Seror et al.	★	★	★	★	★	★	6
Backes et al.	★	★	★	★			4
Bernardini et al.	★	★	★	★	★		5
GEPPERT et al.	★	★	★	★			4
Tang et al.	★	★	★	★	★	★	6
DeNardis et al.	★	★	★	★	★		5

Supplementary Table 2. The quality of included observational study assessed by the Newcastle-Ottawa scale

Study	Selection				Comparability	Outcome			Quality score
	Representativeness of the exposed cohort	Selection of the non-exposed cohort	Ascertainment of exposure	Demonstration that outcome of interest was not present at start of study	Comparability of cohorts on the basis of the design or analysis	Assess ment of outcom e	Was follow-up long enough for outcomes to occur	Adequacy of follow up of cohorts	
Lim et al. (2011)	★	★	★	★	★★	★	★		8
Eklind et al.	★	★	★	★	★★		★		7
Lim et al. (2010)	★	★	★	★	★★	★	★		8
Seamon et al.	★	★	★	★	★★	★	★		8
JUNG et al.	★	★	★	★	★★		★		7
Estape et al.	★	★	★	★	★★		★		7
Veljovich S et al.	★	★	★	★	★★		★		7
Manchana et al.	★	★	★	★	★	★	★	★	8
Coronado J et al.	★	★	★	★	★	★	★	★	8
Corrado et al.	★	★	★	★	★	★	★	★	8
Goicoechea et al.	★	★	★	★	★	★	★	★	8

Magrina et al.	★	★	★	★	★		★	★	7

Supplementary Table 3. The surface under the cumulative ranking curve values of four surgical methods for all outcomes

Groups	Disease Free Survival	Overall survival	Pelvic lymph nodes	Para-aortic lymph nodes	Intraoperative complications	Postoperative complications	Operative time
OH	0.56	0.52	0.89	0.54	0.73	0.01	0.97
LH	0.81	0.87	0.42	0.62	0.26	0.55	0.32
RH	0.21	0.26	0.05	0.12	0.56	0.95	0.66
LAVH	0.41	0.35	0.65	0.72	0.45	0.49	0.05

Abbreviations: OH, open hysterectomy; LH, laparoscopic hysterectomy; RH, robotic hysterectomy; LAVH, laparoscopic-assisted vaginal hysterectomy.

Supplementary Table 4. Egger test for operative time and pelvic lymph nodes

Outcomes	95% confidence intervals	P value
Operative time	-2.37,3.95	0.61
Pelvic lymph nodes	-3.51,4.03	0.89

Supplementary Table 5. The surface under the cumulative ranking curve values of five surgical methods in subgroup analysis

Groups	Disease Free Survival
OH	0.45
LH-U	0.34
LH-NU	0.62
LAVH-U	0.52
LAVH-NU	0.55

Abbreviations: OH, open hysterectomy; LH-U (laparoscopic hysterectomy use of uterine manipulator), LH-NU (laparoscopic hysterectomy non-use of uterine manipulator), LAVH-U (laparoscopic-assisted vaginal hysterectomy use of uterine manipulator), and LAVH-NU (laparoscopic-assisted vaginal hysterectomy non-use of uterine manipulator).

Supplementary Table 6. Meta-regression of confounding covariates influencing heterogeneity

Outcomes	Covariates	β, P value<br>A *vs.* B
Operative time	Publication Years (before 2010/ After 2010)	-8.26, 0.70
	Countries (USA / non-USA)	4.37, 0.85
	Publication types (RCT / non-RCT)	-2.6, 0.91
Pelvic lymph nodes	Publication Years (before 2010/ After 2010)	-0.87, 0.78
	Countries (USA / non-USA)	-3.70, 0.20
	Publication types (RCT / non-RCT)	-3.05, 0.34

Abbreviations: β, regression coefficient. A, open hysterectomy (OH); B, laparoscopic hysterectomy (LH).
