# Supplementary material for: Different surgical methods of hysterectomy for the management of endometrial cancer: a systematic review and network meta-analysis
Source: Front Oncol. 2025 Jan 15;14:1524991. doi: 10.3389/fonc.2024.1524991 (PMC11774694; doi:10.3389/fonc.2024.1524991)
Supplement: Supplementary file 2 [file DataSheet2.docx]

**Supplementary appendix**

**Supplementary Figure 1.** Cochrane system bias evaluation of included randomized controlled trials.

**Supplementary Figure 2.** Network relationship plots of four surgical methods for all outcomes

The size of each circle corresponded to the number of patients, and the thickness of the lines between the two surgical methods reflected the number of studies.

Abbreviations: OH, open hysterectomy; LH, laparoscopic hysterectomy; RH, robotic hysterectomy; LAVH, laparoscopic-assisted vaginal hysterectomy.

**Supplementary Figure 3.** Cumulative ranking plots and rank nomogram of four surgical methods

A. Disease-free survival and higher rankings associated with smaller values. B. Overall survival and higher rankings associated with smaller values. C. The number of retrieved pelvic lymph nodes and higher rankings associated with larger values. D. The number of retrieved para-aortic lymph nodes and higher rankings associated with larger values. E. Incidence of intraoperative complications and higher rankings associated with smaller values. F. Incidence of postoperative complications and higher rankings associated with smaller values. G. Operative time and higher rankings associated with smaller values.

Abbreviations: OH, open hysterectomy; LH, laparoscopic hysterectomy; RH, robotic hysterectomy; LAVH, laparoscopic-assisted vaginal hysterectomy.

**Supplementary Figure 4.** Leverage plots of four surgical methods

Abbreviations: pD, effective number of parameters; Dres, deviation of total residual; DIC, deviance information criterion.

**Supplementary Figure 5.** Posterior mean deviance comparison plots of four surgical methods

Each data point showed a treatment arm's contribution to the posterior mean deviance for the consistency model (horizontal axis) and the inconsistency model (vertical axis).

**Supplementary Figure 6.** Funnel plots of four surgical methods for assessing the publication bias

In the comparison-adjusted funnel plot, the horizontal axis shows the difference of each i-study estimate YiXY from the summary effect for the respective comparison (YiXY-μXY) while the vertical axis presents the measure of dispersion of YiXY, namely the standard error of the effect size. Each point represents a direct comparison; different colors correspond to different comparisons. The dashed black line represents the 95% confidence interval. Abbreviations: OH, open hysterectomy; LH, laparoscopic hysterectomy; RH, robotic hysterectomy; LAVH, laparoscopic-assisted vaginal hysterectomy.

**Supplementary Figure 7.** Heat plots of the league table for the five surgical methods in subgroup analysis

Abbreviations: OH, open hysterectomy; LH-U (laparoscopic hysterectomy use of uterine manipulator), LH-NU (laparoscopic hysterectomy non-use of uterine manipulator), LAVH-U (laparoscopic-assisted vaginal hysterectomy use of uterine manipulator), and LAVH-NU (laparoscopic-assisted vaginal hysterectomy non-use of uterine manipulator).

**Supplementary Table 1.** The quality of excluded observational study assessed by the Newcastle-Ottawa scale

**Supplementary Table 2.** The quality of included observational study assessed by the Newcastle-Ottawa scale

**Supplementary Table 3.** The surface under the cumulative ranking curve values of four surgical methods for all outcomes

Abbreviations: OH, open hysterectomy; LH, laparoscopic hysterectomy; RH, robotic hysterectomy; LAVH, laparoscopic-assisted vaginal hysterectomy.

**Supplementary Table 4.** Egger test for operative time and pelvic lymph nodes

**Supplementary Table 5.** The surface under the cumulative ranking curve values of five surgical methods in subgroup analysis

Abbreviations: OH, open hysterectomy; LH-U (laparoscopic hysterectomy use of uterine manipulator), LH-NU (laparoscopic hysterectomy non-use of uterine manipulator), LAVH-U (laparoscopic-assisted vaginal hysterectomy use of uterine manipulator), and LAVH-NU (laparoscopic-assisted vaginal hysterectomy non-use of uterine manipulator).

**Supplementary Table 6.** Meta-regression of confounding covariates influencing heterogeneity

Abbreviations: β, regression coefficient. A, open hysterectomy (OH); B, laparoscopic hysterectomy (LH).
